# Transovarial transmission of Yersinia pestis in its flea vector, Xenopsylla cheopis

**DOI:** 10.21203/rs.3.rs-3397969/v1

**Published:** 2023-10-27

**Authors:** Deborah Anderson, Cassandra Pauling, Brenda Beerntsen, Qisheng Song

**Affiliations:** University of Missouri; University of Central Missouri; University of Missouri; University of Missouri

**Keywords:** flea, Xenopsylla, Yersinia, plague, transovarial transmission

## Abstract

*Yersinia pestis* is the causative agent of bubonic plague, a deadly flea-borne disease responsible for three historic pandemics. Today annual cases of human disease occur worldwide following exposure to *Y*. *pestis* infected fleas that can be found within the rodent population where plague activity cycles between epizootic outbreaks and extended periods of apparent quiescence. Flea transmission of *Y. pestis* is most efficient in “blocked” fleas that are unable to feed, whereas mammalian transmission to fleas requires a susceptible host with end-stage high titer bacteremia. These facts suggest alternative mechanisms of transmission must exist to support the persistence of *Y. pestis* between epizootic outbreaks. In this work, we addressed whether vertical transmission could be a mechanism for persistent low-infection across generations of fleas. We demonstrate that *Y. pestis* infection of the Oriental rat flea, *Xenopyslla cheopis*, spreads to the reproductive tissues and is found in eggs produced by infected adult fleas. We further show that vertical transmission of *Y. pestis* from eggs to adults results in midgut colonization indicating a strong probability that it can reenter the sylvatic plague cycle.

## Summary

Risk of plague, caused by *Yersinia pestis*, exists in endemic areas present in four continents due to the stable transmission cycle between rodents and their associated fleas ^1^. Plague cycles between quiescent phases that may last decades and explosive epizootic outbreaks that increase human exposure ^2–6^. Acquisition and regurgitative transmission of *Y. pestis* by fleas are inefficient mechanisms, associated with a shortened lifespan of the flea or rodent host ^7,8^. Collectively, these features are seemingly incompatible, indicating that, despite over 100 years of intensive research on flea transmission of plague, there remain significant gaps in our understanding of the vector-animal cycle of *Y. pestis*. Herein, we found that vertical transmission is a missing gap in knowledge which can account for sustained *Y. pestis* infection of a flea population. We demonstrate that laboratory-reared and infected plague vector, *Xenopsylla cheopis*, transmits viable *Y. pestis* from adults to eggs that is subsequently successfully passed through all life stages. Further evidence indicates that transovarial transmission occurs throughout the life cycle of the infected flea. Together these data implicate transovarial transmission as a mechanism capable of sustaining *Y*. *pestis* in the environment for extended periods without detectable plague activity.

## Introduction

*Yersinia pestis* is the causative agent of plague, which is classified as a prioritized re-emerging zoonotic disease within the United States. Worldwide plague foci persist due to enzootic sylvatic cycles consisting of burrowing rodents and their respective flea vectors, which are a crucial component for transmission ^9–15^. Numerous flea species and primarily rodent hosts are implicated in maintaining the sylvatic plague cycle, with mice, rats, prairie dogs, and ground squirrels commonly found in established plague foci ^16,17^. Fleas can transmit *Y. pestis* as early as day 1 post-infection, in a poorly understood mechanism involving regurgitation from the midgut with an enhanced efficacy that results from high flea density ^18^. Once in the midgut, bacteria respond to environmental cues that activate expression and production of an exopolysaccharide matrix, which develops into an infectious biofilm ^19,20^. During feeding the biofilm localized to the proventriculus occludes ingestion and causes regurgitation which deposits *Y. pestis* in the dermis of the human or animal host. From this site, as few as one bacterium is sufficient to cause lethal bubonic plague.

Early studies of the rodent-flea plague cycle documented proventricular blockage and its potential Correlation to transmission in fleas known to vector *Y. pestis*. Blockage and transmission by blocked fleas was most frequent in the rat flea, *Xenopsylla cheopis*, with more than 50% occurrence, whereas blockage was very infrequent in other plague vectors including *Oropsylla montana* ^21^. Recent work demonstrated that occurrence of proventricular blockage in *Y. pestis*-infected *O. montana* could be enhanced by using rat blood feeding compared to mouse blood ^22^. This observation suggests that increased populations of rats should increase the likelihood of epizootic plague, however this is not observed in naturally occurring plague foci. In addition to biofilm-mediated proventricular blockage, *Y*. *pestis* can be transmitted through a regurgitative mechanism shortly after the flea becomes infected ^23^. This mechanism appears to be independent of blood meal source, but can also result in non-productive transmission suggesting it may slow epizootic spread of disease ^24^. Although these two methods of transmission are believed to require distinct proteins from the bacteria and fleas, both occur from the midgut. There have been no reports of *Y. pestis* localization to salivary glands, and no indication that bacteria are able to escape the midgut of fleas. Thus, the collective data led to the prevailing model for *Y. pestis* transmission involving continuous cycling between adult fleas and susceptible rodent hosts. With these parameters, the persistence of *Y. pestis* in the environment where there is little to no plague activity cannot be modeled.

In this work, we therefore addressed the historic paradigm that *Y. pestis* is confined to the midgut and alimentary canal in fleas with the underlying hypothesis that vertical transmission in the flea could account for long periods of reduced plague activity in a given endemic area. Using a highly sensitive fluorescent reporter with confocal microscopy and additional imaging with transmission electron microscopy, we reexamined the distribution of *Y. pestis* following membrane feeding of *X. cheopis* in male and female fleas and their progeny.

## Results

### Yersinia pestis escapes to reproductive tissue in the flea vector Xenopsylla cheopis.

**Figure 1:**
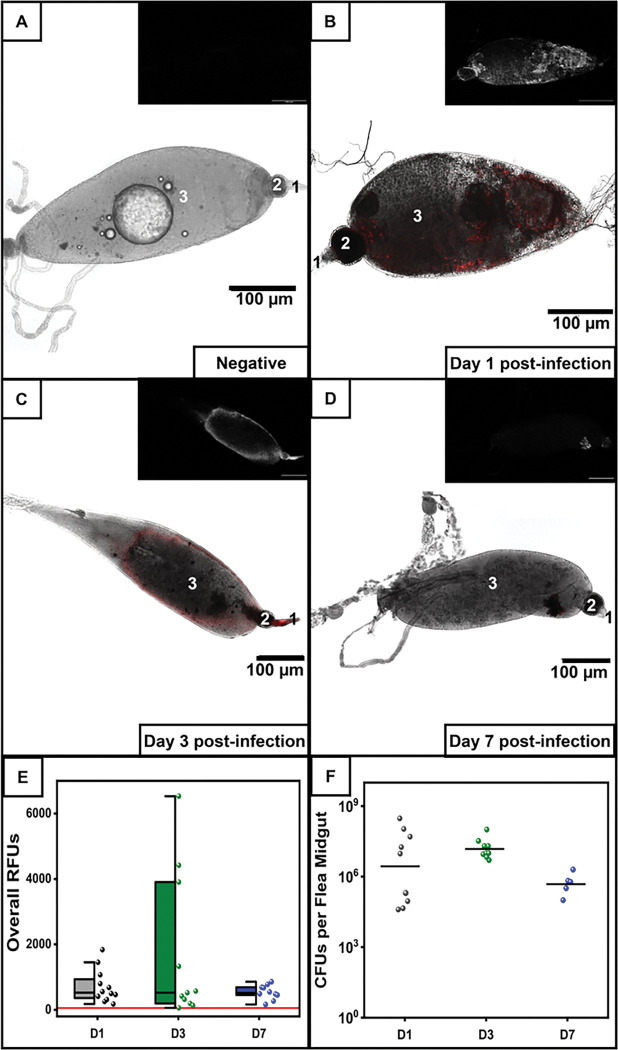
Yersinia pestis expressing tdTomato forms aggregates in the midgut of Xenopsylla cheopis. *X. cheopis* fleas were infected with *Yersinia pestis* in rat blood. (A-D) Representative confocal microscope images of dissected midguts from control adult fleas (A) or (B-D) infected, harvested 1 (B), 3 (C), or 7 (D) days post-infection; inset shows saturation channel only of the same flea. Each image is numbered to show the esophagus (1), proventriculus (2) and midgut (3). (E) Relative fluorescent units (RFU) localized in the alimentary canal; red line shows background RFU; box plots represent 25–75% interquartile range, line indicates median value, whiskers indicate minimum and maximum values; individual data points are shown on right, n=12 (D1), n=11 (D3), n=10 (D7). (F) Bacterial load (colony forming unit, CFU) in the dissected midguts determined in pools of 3 midguts per data point, n=9 pools of 3 (D1, D3), n=5 pools of 3 (D7). Data shown were collected in 3 independent trials.

To understand *Y. pestis* interactions in fleas, we used a highly sensitive expression system for the fluorescent reporter tdTomato as a means to localize bacteria following infection of *X. cheopis* fleas in an artificial membrane feeder. With this approach, we established the behavior of fluorescent *Y. pestis* in the midgut using confocal microscopy and followed an apparent *Y. pestis*-containing biomass that developed and peaked in the midgut on day 3 post-infection (Fig. 1A–D). In some of the fleas collected mainly on day 3, brightly fluorescing *Y. pestis* could be seen in the esophagus, similar to previous reports (representative shown in Fig. 1C) ^22,25^. By day 7, the *Y. pestis* containing biomass decreased, consistent with previous studies (Fig. 1D–E). The kinetics of the biomass suggests correlation with competency for early phase transmission. In parallel, bacterial load was quantified in pools of 3 midguts per group, demonstrating similar median values for colony forming units (CFU) across the 7-day time course (Fig. 1F). These data establish that while the kinetics of the *Y. pestis*-containing biomass change during the 7-day course of infection, viable bacterial load is relatively constant in the flea midgut.

**Figure 2:**
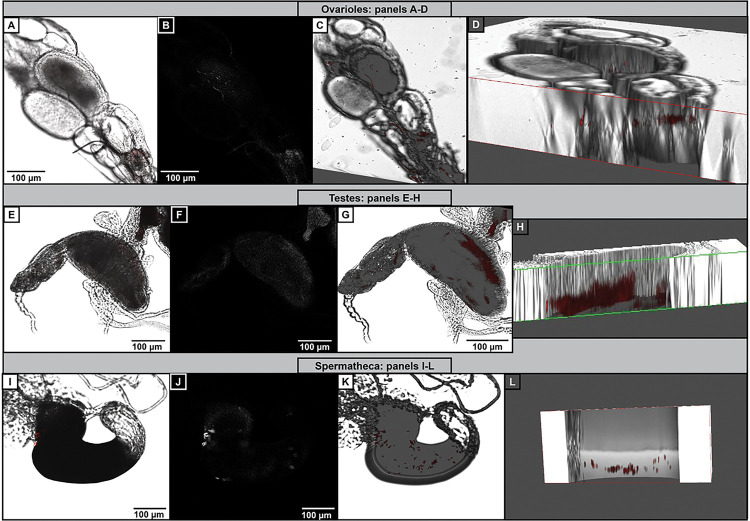
Bacteria is present in reproductive tissues during infection with different host bloodmeals. Representative images from individual adult fleas that were infected with *Y. pestis* KIM6+ptdTomato in an artificial membrane feeder. (A-D) Ovarioles dissected from day 7 post-infection using prairie dog blood and imaged by confocal microscopy: (A) RGB image; (B) Saturation channel; (C-D) 3D rendition of the ovarioles to show the bacteria within the tissues. (E-H) Testes dissected day 5 post-infection using rat blood, imaged by confocal microscopy: (E) RGB image; (F) Saturation channel; (G-H) 3D rendition of the testes to show the bacteria within the tissues. (I-L) Spermatheca from day 5 following infection with pig bloodmeal and imaged by confocal microscopy: (I) RGB image; (J) Saturation channel; (K-L) 3D image and a cross section. Images were selected from a pool of 10 (A-D), 9 (E-H), and 9 (I-L) individual fleas analyzed from 3 independent trials.

To determine whether *Y. pestis* may be localized outside of the midgut, we examined the ovarioles and the testes of fleas dissected from the various times post-infection. Using confocal microscopy, we generated z-stacks to visualize the inside of the ovarioles and testes. Indeed, *Y. pestis* was readily observable, appearing to localize inside the ovarioles rather than on the outside surface (Fig. 2A–D). Similarly, we readily observed tomato-fluorescing *Y. pestis* in the testes of male adult fleas (Fig. 2E–H). Therefore, perhaps not unexpectedly, *Y. pestis* was also found in the spermatheca (Fig. 2I–L). Other tissues that contained *Y. pestis* that was visible by confocal microscopy included Malpighian tubules, but not salivary glands (Extended Data Fig. 1, salivary glands not shown). Furthermore, *Y. pestis* could be seen in the reproductive organs up to 15 weeks after the adult fleas were infected (Extended Data Fig. 2). This result suggests that *Y. pestis* infection of the reproductive tissue is stable throughout the lifespan of the adult flea.

### Viable Y. pestis is present in the oviposited eggs and larvae derived from infected adult fleas.

**Figure 3:**
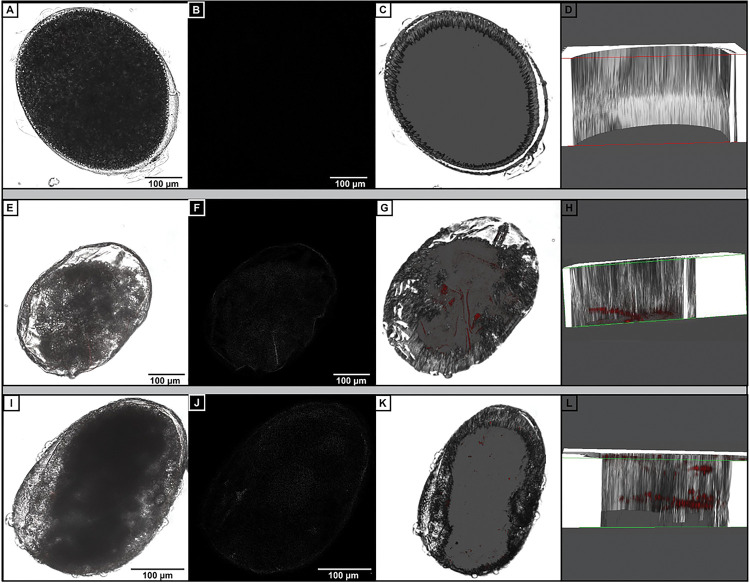
Yersinia pestis is transovarially transmitted in fleas. Eggs were collected from adult *X. cheopis* that were fed uninfected (A-D) or *Y. pestis*-infected (E-L) blood, then processed for confocal microscopy: (A, E, I) Left column contains images from the brightfield/RGB channel; (B, F, J) Associated saturation channel; (C, G, K) 3-D projections of Z-stacks; (D, H, L) Cross section of the 3-D projection to illustrate the bacteria within the egg rather than on its surface. (A-H) were collected day 3 post-blood feeding/infection; (I-L) were collected day 7 post-infection. Images were selected from a pool of 14 eggs collected from 4 independent trials.

To determine whether viable *Y. pestis* was present in the eggs, we devised an egg collection chamber, using sifting to separate the eggs from bedding debris. Adult fleas that are feeding regularly have a steady reproduction at 3.6 to 5.6 eggs per female per day which decreases as the fleas age ^26,27^. Eggs from *X. cheopis* are 477–504 μm long with a width of 297–333 μm and off-white in color. Sifting was carried out every 1–3 days and eggs were collected of the expected size and color. Eggs were thoroughly washed and then processed for imaging or for plating to determine bacterial titer. Control fleas, given blood that was not infected, produced eggs with no fluorescent signal either on or within the egg tissue (Fig. 3A–D). In sharp contrast, tdTomato fluorescence was abundant in the interior of eggs produced by *Y. pestis*-infected fleas, localizing to nearly the length of the egg (Fig. 3E–L). We found prominent fluorescent signal in more than half of the eggs that were tested, whether they were collected 3 (Fig. 3D–F) or 7 (Fig. 3I–L) days post-infection. Considering blood intake initiates copulation and subsequent oviposition, the data suggest that egg-derived *Y. pestis* were either transferred directly to the egg due to midgut escape during the bloodmeal, or that colonization of the spermatheca results in the continuous opportunity for *Y. pestis* to infect newly developing eggs ^28^.

**Figure 4:**
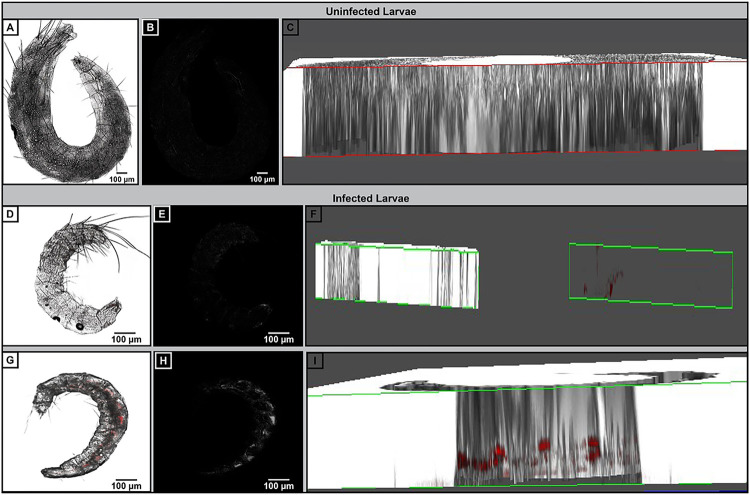
Yersinia pestis is transstadially transmitted in X. cheopis. Larvae were collected from *X. cheopis* adult fleas fed in an artificial membrane feeder from uninfected (A-C) or *Y. pestis*-infected (D-I) blood, then processed for confocal microscopy. Representative images from individual larva are shown: (A, D, G) RGB images with associated conversion to HSB; (B, D, H) Associated saturation channel along with 3-D cross section (C, F, I) are shown. Infected larvae were either collected from the same housing as the infected adults (D-F) or after separating the eggs from infected adults and hatching in fresh, sterile housing (G-I). Images were selected from a pool of 9 larvae collected from 3 independent trials.

We also collected larvae by sifting and found 5 of 8 were positive, with tdTomato fluorescence that appeared to be present in nearly the entire length of the larvae (Fig. 4D–I) compared to larvae from control fleas which were uninfected (Fig. 4A–C). To rule out the likelihood that larvae were infected by eating *Y. pestis* that had been expelled from the hindgut, we collected eggs that were deposited by *Y. pestis*-infected adult *X. cheopis* and reared them in a separate sterile container into larvae. As expected, fluorescent *Y. pestis* was readily visualized in these samples (Fig. 4G–I). Together, these data indicate that *Y. pestis* in the eggs survives development into the larval stage. Additional eggs from *Y. pestis*-infected adult fleas were adults. Pupae exhibited a high degree of autofluorescence (Extended Data Fig. 3A-B). Nevertheless, strong tdTomato expression could be detected in the pupae reared from *Y. pestis*-infected eggs (Extended Data Fig. 3C-D). Likewise, fluorescent *Y. pestis* could also be visualized in the midguts of the F1 adult fleas (Extended Data Fig. 4A-D). Bacteria harvested from these fleas were screened for *Y. pestis* plasmid-encoded genes, *caf1* and *pla* by PCR, and indeed, the transovarially-transmitted bacteria were positive for both genes, suggesting they retained the extrachromosomal plasmids (Extended Data Fig. 4E).

### Transovarially acquired Y. pestis is competent for transmission.

**Figure 5:**
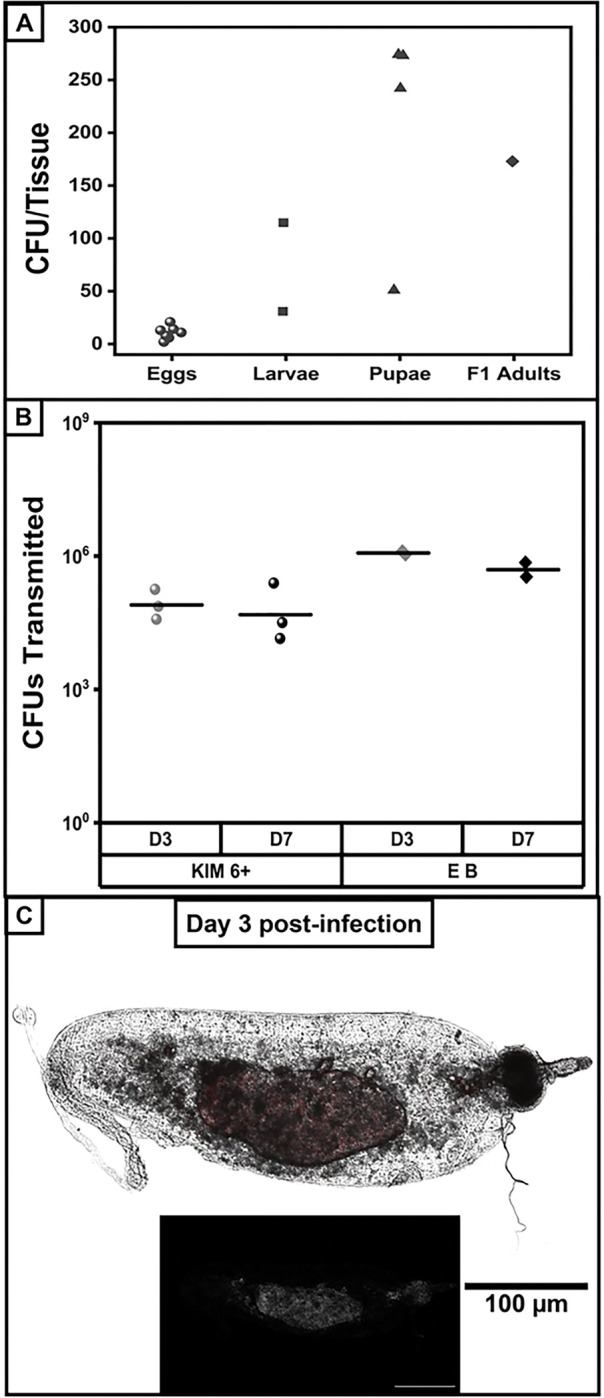
Transstadially transmitted Yersinia pestis retains plasmids and capability for transmission (A) Eggs and larvae were collected from *Y. pestis*-infected *X. cheopis* fleas and either processed or reared to pupae and F1 adult fleas. Samples were processed to quantify bacterial load; F1 adult midguts were pooled (n=2), n=7 eggs, n=2 larvae, n=4 pupae. (B-C) *Y. pestis* isolated from eggs collected from infected adults was used to infect naïve *X. cheopis* fleas. On days 3 and 7 post-infection, a subset of infected fleas was used in a transmission assay in an artificial membrane feeder; for day 3 transmission, blood was collected from the feeder and plated to quantify bacterial load (B) or for fluorescence microscopy of the infected midgut (C). Data shown were collected in two independent trials with n=10 fleas and n=7 fleas for day 3, and n=8 fleas and n=7 fleas for day 7.

To determine whether transovarially transmitted bacteria were viable, we compared bacterial titers in eggs, larvae, pupae and F1 progeny. The median number of colonies recovered from eggs was only 5 CFU (Fig. 5A). In the later stages of development, bacteria appeared to replicate, with median titer recovered in larvae of approximately 50 CFU, and in pupae, the median titer was over 100 CFU. In the newly emerged adult F1 fleas collected prior to their first blood feeding, two midguts were harvested and combined for plating, resulting in 173 CFU recovered. To confirm the retention of extrachromosomal plasmids, bacterial stocks were made and then used for PCR to amplify the *Y. pestis* plasmid-specific genes *caf1* and *pla*. Following PCR, the expected-size bands were purified and processed to determine the DNA sequence in order to conclusively confirm *Y. pestis* (Extended Data Fig. 4E, sequence data not shown). Together, these data indicate that transovarially transmitted *Y. pestis* remain viable throughout the developmental cycle and the recovery of viable *Y. pestis* from the midgut of F1 adult fleas suggests that transovarially transmitted bacteria are competent for transmission.

We therefore evaluated whether *Y. pestis* isolated from eggs remained competent for flea transmission to a mammalian host. Naïve adult fleas were challenged with egg-isolated *Y. pestis* in the artificial membrane feeder and on days 3 and 7, infected fleas were used in a transmission study. On day 3 post-infection, it was evident that egg-isolated *Y. pestis* had been successfully transmitted, and its localization to midgut biomass appeared indistinguishable from fleas infected with the original *Y. pestis* stock (Fig. 5B–C). Similar kinetics of egg-isolated *Y. pestis* were observed in the flea midgut as seen for the parent *Y. pestis* strain with similar bacterial load observed on day 7 post-infection. Overall, these data suggest that transovarially transmitted *Y. pestis* is competent for re-entry into the sylvatic plague cycle.

### Transmission electron microscopy (TEM) shows Y. pestis localized to reproductive tissue in adult fleas.

**Figure 6:**
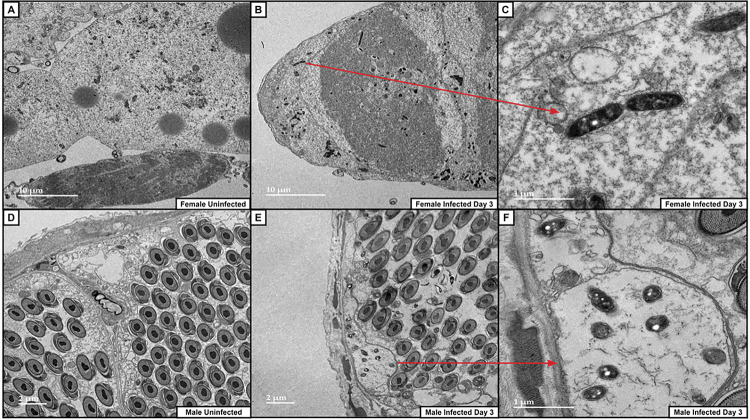
Yersinia pestis colonizes ovaries and testes of infected adult X. cheopis. Ovaries and testes were dissected from *X. cheopis* on day 3 following bloodmeal infection with *Y. pestis* (B, C, E, F); control fleas fed with sterile rat blood (A, D). Ovaries from female control (A) or infected (B-C); red arrow in B indicates the section where a zoomed-in image was taken, pointing to replicating *Y. pestis* in the “safety pin” morphology. Testes from male control (D) or infected (E-F) adult fleas showing numerous bacteria within an enlarged basement membrane; arrow points to section where zoomed-in image was taken.

To confirm that bacteria were present in the testes and ovaries of infected adult fleas, we used transmission electron microscopy to image tissues that were dissected from male and female fleas. In the females, we observed bacteria in the ovaries, and even in the safety pin morphology that is characteristic of *Y. pestis* (Fig. 6B–C). These bacteria were not found in fleas that were not infected (Fig. 6A). In the male fleas, we readily identified abundant *Y. pestis* in the basal membrane of the testes, appearing on the distal side of the spermatocyte follicle, often within membrane-enclosed vesicles (Fig. 6D–F). This suggests that *Y. pestis* may be intracellular in the reproductive tissue. Overall, the TEM confirms that *Y. pestis* exit the midgut and localize to the reproductive organs of an adult flea.

## Discussion

In this work, we have provided multiple layers of evidence that transovarial transmission occurs in *X. cheopis* infected with *Y. pestis*. These findings provide visual, quantitative, and genetic support that low levels of *Y. pestis* escape from the midgut throughout the lifespan of an infected flea, entering the reproductive tract where they are able to colonize progeny eggs. In the more than 100 years of *Y. pestis* research, escape from the alimentary tract has never been reported. Previous studies were heavily focused on salivary glands, and in agreement with these results, we did not observe any *Y. pestis* in the salivary glands. Furthermore, the amount of colony forming units harvested from flea eggs is exceedingly small, averaging fewer than 10 CFU, consequently it may simply be that previous experiments were not sensitive enough to capture the low level of transovarial transmission. In this work, the high-level expression of the tdTomato reporter revealed a new understanding of sylvatic plague.

Fleas are vectors of other diseases, namely rickettsiosis and cat scratch fever caused by *Rickettsia felis* or *typhi* or *Bartonella henselae*, respectively. For *Rickettsia typhi*, transovarial transmission was observed in fleas more than 30 years ago ^29^. Like *Rickettsia felis*, intracellular *Y. pestis* appear to localize to the ovariole tissue ^30^. However, there are likely significant differences in the underlying mechanism of midgut escape. Whereas *R. felis* infection of the midgut epithelium was readily apparent shortly after infection, there was no detectable infection of these cells by *Y. pestis*. Following invasion of the midgut epithelium *R. felis* was observed in hemocoel prior to its systemic dissemination to the reproductive tract and salivary glands. In contrast, *Y. pestis* was not observed in hemocoel or salivary glands.

Transovarial transmission could be a contributor to maintenance of *Y. pestis* within the environment. From the data shown here, viable *Y. pestis* is continuously deposited in eggs, suggesting most, if not all, progeny fleas remain infected. Furthermore, the strong selective pressure of the sylvatic plague cycle would favor the retention of virulence factors by transovarially-transmitted *Y. pestis*. Therefore, it seems likely that transovarial transmission could sustain low levels of *Y. pestis* in a rodent community. Environmental factors such as precipitation and temperature are predicted to have an impact on the transovarial transmission cycle, thereby having multi-layered contributions to the prevalence of *Y. pestis* in the environment. By understanding transovarial transmission, we may improve our ability to model the enzootic cycle of plague.

## Methods

### Animals:

Neonatal and adult mice were used in these experiments for colony maintenance blood feeding as well as preparation of the artificial feeder, according to previously published protocols ^31,32^. Colony fleas were provided opportunity for blood feeding 2–3 times/week prior. The blood feeding and infection protocol were approved by the University of Missouri Animal Care and Use Committee.

### Infection:

These experiments were conducted with a laboratory strain of *Yersinia pestis* KIM6+, which lacks the type III secretion system plasmid pCD1 ^33^. The recombinant derivatives of KIM6+ used in this work did not reintroduce the pCD1 plasmid, and therefore are classified as select agent-exempt strains by the US Center for Disease Control and Prevention. Prior to use in infection, laboratory reared, naive *X. cheopis* were separated from the colony and starved for at least five days and no more than seven days to improve feeding efficiency during infection. Groups of 50 fleas were infected with *Y. pestis* strain KIM6+ carrying plasmid-expression of the fluorescent protein tdTomato (Excitation: 554, Emission 581) ^34^. An artificial membrane feeder was constructed using skin from an adult mouse. Blood was inoculated with 5×10^8^ to 1×10^9^ *Y. pestis* and maintained at 37°C, and fleas were allowed to feed for 1 hour. The species of animal blood (rat, mouse, pig, or prairie dog) used in the artificial feeder is indicated in the figure legends. When fleas were removed from the feeder, they were observed to determine intake of the bloodmeal. Fleas that had not fed were removed from the study.

### Midgut processing:

Fleas were euthanized on days 1, 3 and 7 post-infection without additional blood feeding. For experiments that lasted more than 7 days, fleas were provided maintenance blood meal every 7 days throughout the duration. Dissected midguts and other reproductive tissues were isolated and placed onto sterile slides, fixed with 4% paraformaldehyde, and rinsed 3 times with PBS for a minimum of 30 minutes each time prior to mounting.

### Confocal microscopy:

Midguts, eggs, larvae and pupae were imaged using a Leica SP8 confocal microscope. For quantification of midgut biomass, images were converted to 8-bit grayscale for quantification of observed integrated density (ID_O_). Control fleas (n=10), fed in parallel with uninfected blood and analyzed 1 day after feeding, were used to determined background fluorescence. Background signal and midgut area were used to normalize the samples and calculate integrated density (ID= IDO − (midgut area × background)). Image J software was used to capture images and quantify the signal intensity, reported as relative fluorescent units (RFU) ^35^.

### Quantification of bacterial load:

For plating, individual samples were homogenized in 10μL sterile PBS, midguts were pooled in groups of 3; serial dilutions were performed in sterile PBS and all dilutions were plated in duplicate onto heart infusion agar (HIA) or *Yersinia* selective agar (YSA). Isolated colonies from eggs, larvae, pupae, and F1 adults were streaked for isolation before storing in bacteriological freezing media at −80°C.

### Egg collection:

Infected fleas using rat blood were maintained in modified housing with 300-micron mesh on the lid, 550-micron mesh under the bedding, and a lower chamber that could be easily removed. Eggs and larvae were collected by sifting, with care to avoid contact with infected feces and fleas. Fleas were maintained in the modified container housing and provided uninfected rat bloodmeal every 7 days. Sifting for eggs and larvae occurred every 1–3 days post-infection. To aseptically collect the eggs and larvae, a sterilized cotton applicator was moistened with double-distilled, sterile H_2_O and used to pick up eggs or larvae. These specimens were placed onto a sterile slide, washed in sterile PBS three times, observed between washing to ensure no materials or feces were in contact, then mounted in 35% glycerol for confocal microscopy or transferred to sterile PBS. After washing three times in sterile PBS, a new sterile, moistened cotton applicator was used to transfer the egg to an agar plate. Each egg was punctured with a sterile needle, releasing the contents onto the agar. Similarly, larvae were washed three times with sterile PBS, then homogenized and plated.

### Development of Y. pestis-infected eggs or larvae:

Eggs were isolated, washed in sterile PBS, and placed into a sterile flea chamber with mesh on the top and bottom containing sterile sawdust and larval food. These samples were imaged or plated on agar after development into larvae, pupae, or F1 adults.

### PCR and genome annotation:

Bacteria that were isolated from eggs were grown overnight and DNA was isolated using a *Quick*-DNA^®^ Microprep kit (Zymo Research, California, USA). Conventional PCR amplification of *caf1* and *pla* were performed using the primers shown in Extended Data Table 1. All positive PCRs were confirmed by sequencing; sequenced nucleotides were aligned in Geneious Prime using MAFFT ^36^.

### Transmission study:

Transmission assays were carried out as previously described for fleas infected with KIM6+ptdTomato ^8^. For assays using bacteria originally harvested from eggs, minor modifications were made. Briefly, egg isolated bacteria were used to infect adult fleas on an artificial feeder. On day 3 or 7 post-infection, groups of 10 fleas were used for the transmission assay and fed on uninfected rat bloodmeal in the artificial membrane feeder for 1 hour. Blood and skin were processed to quantify *Y. pestis* by plating to determine the number of bacteria transmitted per group.

### Transmission electron microscopy.

Following the same methods, fleas were either fed an uninfected rat bloodmeal or a *Y. pestis*-infected rat bloodmeal and then groups of 10 were separated based on sex. On day 3, fleas were euthanized, and the ovaries, testes, and midguts were dissected, fixed in 2% paraformaldehyde, 2% glutaraldehyde in 100 mM sodium cacodylate buffer with a pH of 7.35. Each sample was allowed to settle, and the resulting tissue was resuspended in Histogel (ThermoScientific, Kalamazoo, MI). Tissues were rinsed in 100 mM sodium cacodylate buffer with a pH of 7.35 containing 130 mM sucrose. Secondary fixation was performed using 1% osmium tetroxide (Ted Pella, Inc. Redding, California) in cacodylate buffer. Specimens were incubated at 4°C for 1 hour, then rinsed with cacodylate buffer and further with distilled water. En bloc staining was performed using 1% aqueous uranyl acetate and incubated at 4°C overnight, then rinsed with distilled water. A graded dehydration series was performed using ethanol, transitioned into acetone, and dehydrated tissues were then infiltrated with Epon resin and polymerized at 60°C overnight. Sections were cut to a thickness of 75 nm using an ultramicrotome (Ultracut UCT, Leica Microsystems, Germany) and a diamond knife (Diatome, Hatfield PA). Images were acquired with a JEOL JEM 1400 transmission electron microscope (JEOL, Peabody, MA) at 80 kV on a Gatan Rio CMOS camera (Gatan, Inc, Pleasanton, CA). All samples were prepared, processed and imaged at the University of Missouri Electron Microscopy core.

### Statistical analysis.

Data was grouped based on trial and microscopic analysis of individual flea midguts and tissue, with controls groups derived from the parent *Y. pestis* and uninfected fleas. Descriptive statistics were evaluated using SPSS 26 and graphed using OriginPro.

## Data Availability

The sequence chromatographs and alignments, image data sets and corresponding metadata that support the findings of this study are available in Figshare with the identifier 10.6084/m9.figshare.c.6845496. [37] All unique biological materials, including bacterial strains and plasmids, are available from the corresponding author upon request. There are no restrictions on any of these materials.
